# Gastroprotective Effects of Inulae Flos on HCl/Ethanol-Induced Gastric Ulcers in Rats

**DOI:** 10.3390/molecules25235623

**Published:** 2020-11-29

**Authors:** Young-Sik Kim, Ji Hyeon Lee, Jungbin Song, Hocheol Kim

**Affiliations:** Department of Herbal Pharmacology, College of Korean Medicine, Kyung Hee University, 26 Kyungheedae-ro, Dongdaemun-gu, Seoul 02447, Korea; yjbsik@gmail.com (Y.-S.K.); jhjhjh4214@naver.com (J.H.L.)

**Keywords:** *Inula britannica*, gastric ulcer, gastritis, gastric wall mucus, antioxidant, prostaglandin E_2_

## Abstract

Inulae Flos, the flower of *Inula britannica* L., is used as a dietary supplement, beverage, and medicine in East Asia. In this study, we evaluated the gastroprotective effects of Inulae Flos extract (IFE) against gastric mucosal lesions induced by hydrochloric acid (HCl)/ethanol in rats and explored its potential mechanisms by measuring antioxidant enzyme activity, mucus secretion, and prostaglandin E_2_ (PGE_2_) levels. Pretreatment with IFE at doses of 100 and 300 mg/kg significantly inhibited gastric lesions in HCl/ethanol-treated rats. IFE increased the activities of superoxide dismutase and catalase and the levels of glutathione and PGE_2_ in gastric tissues. The administration of IFE also significantly increased the gastric wall mucus contents in HCl/ethanol-induced gastric lesions. These findings suggest that IFE has gastroprotective effects against HCl/ethanol-induced gastric lesions and exerts these effects through increased antioxidant levels and gastric mucus secretion. Inulae Flos may be a promising agent for the prevention and treatment of gastritis and gastric ulcers.

## 1. Introduction

As a common type of upper gastrointestinal disorder, gastric ulcers are induced by an imbalance between aggressive factors (such as hydrochloric acid (HCl) and pepsin secretion) and protective factors (such as prostaglandins, mucus production, antioxidants, and adequate blood flow) [1]. The onset of gastric ulcers is related to not only genetic factors but also environmental factors, such as unhealthy lifestyle (such as excessive alcohol consumption, smoking, and excessive salt intake) and *Helicobacter pylori* infection [2].

The incidence of gastric ulcers is 0.1–0.3%/year, and, in the general population, the lifetime prevalence of this disease is 10% [3]. Although the incidence of gastric ulcers has continued to decline, these ulcers still result in various complications and even death [4]. Antibiotics, H_2_ receptor antagonists, proton pump inhibitors, and antacids have been used to treat gastric ulcers. However, several side effects, including vitamin B12 deficiency, gynecomastia, hypoacidity, osteoporotic fracture, hypergastrinemia, depression, and constipation, have been reported [5,6,7]. Recent studies have identified various natural products as potentially safe alternatives with few side effects [8,9,10].

In our previous study, we screened medicinal herbs that have been used to treat gastritis symptoms in the Korean medicinal literature, using a rat model of gastritis [11,12,13]. During this screening process, the flowers of *Inula britannica* L. (Inulae Flos) were shown to be effective herbs for gastroprotection. *I. britannica*, also known as the British yellowhead, is a perennial herbaceous plant that grows in North America, Europe, and East Asia [14]. In East Asia, Inulae Flos has been traditionally used to treat epigastric fullness, upper abdominal discomfort, and burping, which are representative symptoms of gastric ulcers. Moreover, Inulae Flos has been used as a beverage, dietary supplement, and natural preservative [15,16]. Inulae Flos contains sesquiterpene lactones (e.g., 1,6-*O*,*O*-diacetylbritannilactone, 1-*O*-acetylbritannilactone, neobritannilactone A, and neobritannilactone B) and flavonoids (e.g., luteolin, patuletin, and quercetin) and has been reported to have hepatoprotective, anti-inflammatory, and antitumor effects [14]. However, the specific effects of Inulae Flos on gastric ulcers and the mechanisms mediating these effects have not yet been clarified.

Accordingly, in this study, we evaluated the gastroprotective effects of Inulae Flos extract (IFE) and explored its potential mechanisms, using an HCl/ethanol-induced gastric ulcer model in rats. Because Inulae Flos and components of Inulae Flos have been reported to regulate the activities or levels of antioxidant enzymes and oxidative stress markers, including superoxide dismutase (SOD), catalase (CAT), and glutathione (GSH) peroxidase, as well as malondialdehyde (MDA) in other tissues and cells [17,18,19,20], we hypothesized that Inulae Flos could protect the gastric mucosa through antioxidant mechanisms, including the modulation of SOD, CAT, GSH, and MDA levels. In addition, the effects of Inulae Flos on mucus secretion and the levels of prostaglandin E_2_ (PGE_2_), which plays an important role in the control of acid and mucus secretion and maintenance of mucosal integrity in gastric ulcers [21,22], were investigated.

## 2. Results

### 2.1. High-Performance Liquid Chromatography (HPLC) Analysis of IFE

**Figure 1:**
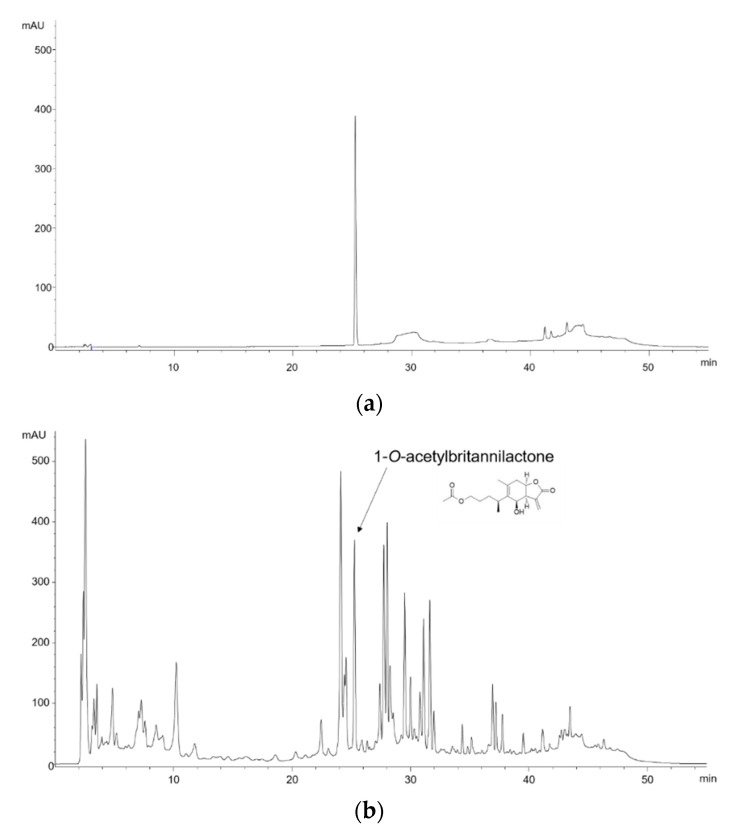
Chromatograms of (**a**) 1-*O*-acetylbritannilactone, the standard compound of Inulae Flos, and (**b**) 95% ethanol extract of Inulae Flos at 210 nm.

Representative HPLC chromatograms of the standard compound and IFE are shown in Figure 1. The content of 1-*O*-acetylbritannilactone in IFE was 16.28 mg/g.

### 2.2. Effect of IFE on HCl/Ethanol-Induced Gastric Injury

**Figure 2:**
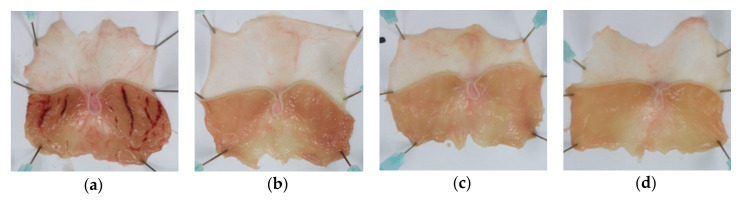
Effects of Inulae Flos extract (IFE) on HCl/ethanol-induced gastric ulcers in rats. Representative gross images of stomachs of (**a**) control, (**b**) Stillen 300 mg/kg, (**c**) IFE 100 mg/kg, and (**d**) IFE 300 mg/kg groups. Rats were treated with distilled water, IFE (100 or 300 mg/kg), or Stillen (300 mg/kg) 1 h prior to the administration of HCl/ethanol.

The administration of HCl/ethanol induced severe hemorrhagic ulcers, as indicated by elongated lines in the glandular region of the stomach (Figure 2). The gastric lesion formation was markedly prevented by pretreatment with IFE and Stillen, a positive control. Furthermore, the gastric lesion area and index were significantly lower in the 100 and 300 mg/kg IFE- and Stillen-treated groups than in the control group (all *p* < 0.001; Table 1). The inhibition rate of IFE against the lesion index was similar to that of Stillen at the same dose of 300 mg/kg.

### 2.3. Histopathological Findings

**Figure 3:**
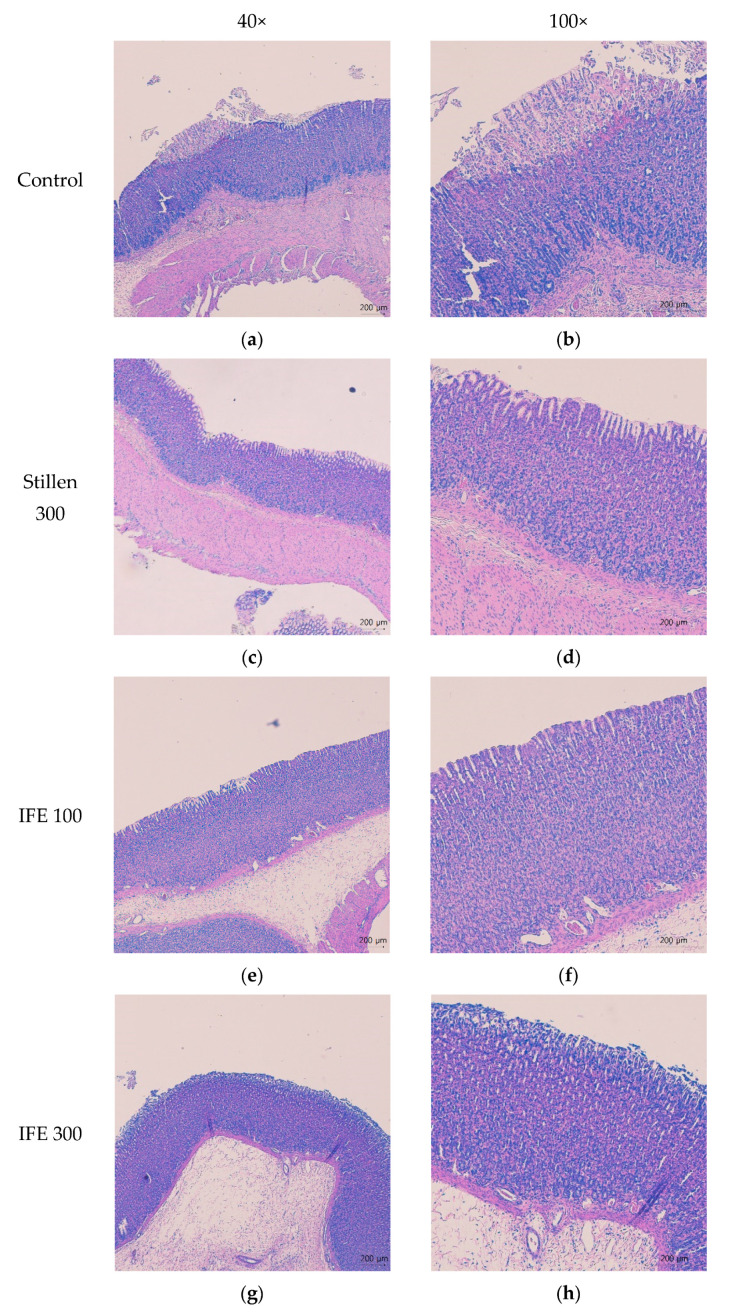
Effects of IFE on histopathological changes in the stomach of rats with HCl/ethanol-induced gastric ulcers. Histopathological changes were analyzed by staining with H&E followed by observation at 40× and 100× magnification (left and right panels, respectively). (**a**,**b**) Control, (**c**,**d**) Stillen 300 mg/kg, (**e**,**f**) IFE 100 mg/kg, (**g**,**h**) IFE 300 mg/kg. Rats were treated with distilled water, IFE (100 or 300 mg/kg), or Stillen (300 mg/kg) 1 h prior to the administration of HCl/ethanol. IFE, Inulae Flos extract.

Rats treated with HCl/ethanol showed marked histological changes in the gastric mucosa with disruption and exfoliation of the superficial gastric epithelium, vacuolization, and necrosis in the superficial mucosal layer associated with gastric lesions (Figure 3a,b). Pretreatment with IFE at doses of 100 and 300 mg/kg and Stillen at 300 mg/kg prevented the congestion and swelling of the gastric mucosal epithelium (Figure 3c–h).

### 2.4. Effects of IFE on SOD and CAT Activities and GSH and MDA Levels

**Figure 4:**
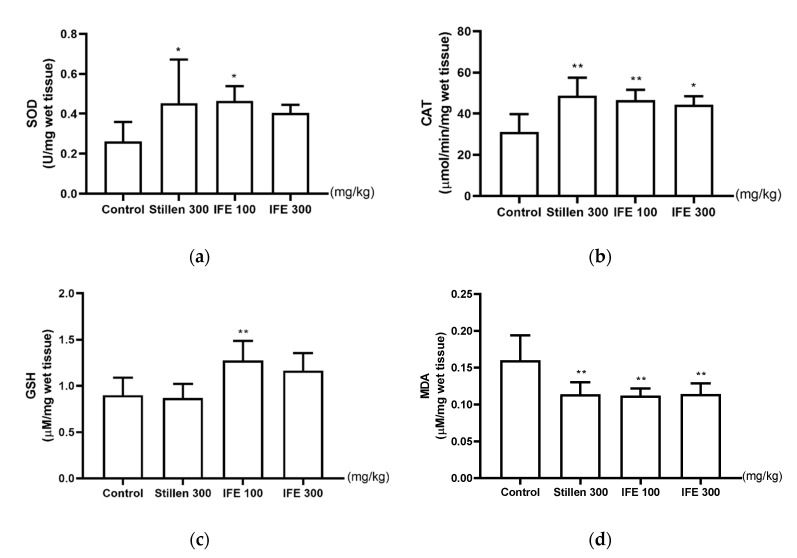
Effects of IFE on (**a**) superoxide dismutase (SOD) and (**b)** catalase (CAT) activities and (**c**) glutathione (GSH) and (**d**) malondialdehyde (MDA) levels in the ulcerated gastric tissue of rats. Rats were treated with distilled water, IFE (100 or 300 mg/kg), or Stillen (300 mg/kg) 1 h prior to the administration of HCl/ethanol. Values are expressed as means ± standard deviations; *n* = 6/group; * *p* < 0.05 and ** *p* < 0.01 versus the control group by one-way analysis of variance with post hoc Dunnett’s test. IFE, Inulae Flos extract.

SOD activity in the stomach was significantly higher in the 100 mg/kg IFE- and Stillen-treated groups than in the control group (both *p* < 0.05, Figure 4a). CAT activity was also higher in the IFE and Stillen groups than in the control group (Figure 4b). GSH levels were significantly higher in the 100 mg/kg IFE group than in the control group (*p* < 0.01, Figure 4c) but were not altered in the 300 mg/kg Stillen-treated group. MDA levels were significantly lower in the 100 and 300 mg/kg IFE- and Stillen-treated groups than in the control group (all *p* < 0.01, Figure 4d).

### 2.5. Effects of IFE on PGE_2_ Concentrations

**Figure 5:**
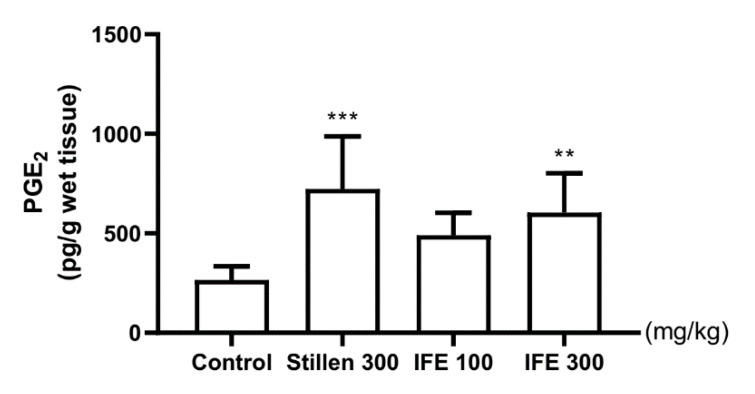
Effects of IFE on prostaglandin E_2_ (PGE_2_) levels in the ulcerated gastric tissue of rats. Rats were treated with distilled water, IFE (100 or 300 mg/kg), or Stillen (300 mg/kg) 1 h prior to the administration of HCl/ethanol. Values are expressed as means ± standard deviations; *n* = 6/group; ** *p* < 0.01 and *** *p* < 0.001 versus the control group by one-way analysis of variance with post hoc Dunnett’s test. IFE, Inulae Flos extract.

The PGE_2_ levels in the ulcerated gastric tissue were significantly higher in the 300 mg/kg IFE- and Stillen-treated groups than in the control group (*p* < 0.01 and *p* < 0.001, respectively; Figure 5).

### 2.6. Effect of IFE on Gastric Wall Mucus Contents

**Figure 6:**
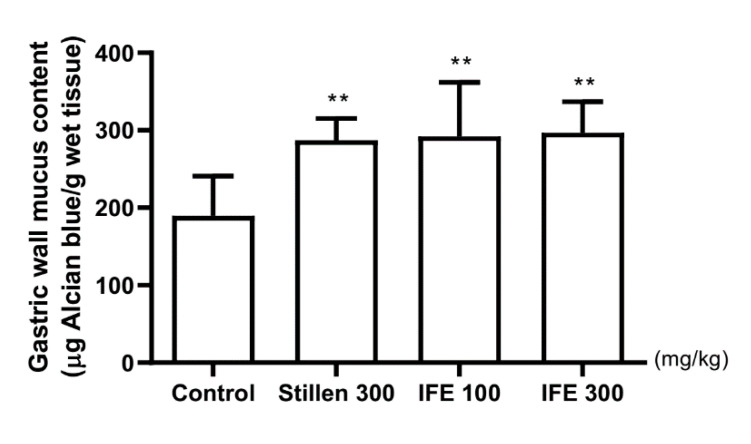
Effects of IFE pretreatment on gastric wall mucus contents in rats with HCl/ethanol-induced gastric ulcers. Rats were treated with distilled water, IFE (100 or 300 mg/kg), or Stillen (300 mg/kg) 1 h prior to the administration of HCl/ethanol. Values are expressed as means ± standard deviations; *n* = 8/group; ** *p* < 0.01 versus the control group by one-way analysis of variance with post hoc Dunnett’s test. IFE, Inulae Flos extract.

The Alcian blue–binding capacity, as a marker of gastric wall mucus content, was 189.60 ± 51.17 μg dye/g tissue in control rats (Figure 6). The capacities were significantly higher, by 51.48%, 54.06%, and 56.59%, respectively, in the 100 and 300 mg/kg IFE- and Stillen-treated groups than in the control group (all *p* < 0.01).

## 3. Discussion

In this study, Inulae Flos pretreatment showed potential gastroprotective effects against HCl/ethanol-induced gastric mucosal damage. Pretreatment with Inulae Flos also increased the amount of adherent mucus and enhanced the antioxidant activity in the gastric mucosa tissues of rats treated with HCl/ethanol.

The gastric mucosa, the superficial layer of gastric tissue, prevents the diffusion of digestive enzymes into the stomach wall by the secretion of gastric mucus [23]. Ethanol penetrates the mucosal layer to the submucosa, causing lesions such as erosion, hemorrhage, and ulcers [24,25]. Ethanol produces reactive oxygen species (ROS) in the gastric mucosa and depletes the mucus layer, causing gastric mucosal cell death [26,27]. Co-treatment with HCl accelerates the damage to stomach tissues [24]. Therefore, the HCl/ethanol-induced gastric ulceration rodent model is commonly used for the evaluation of gastric protective agents as a reproducible and stable method of inducing gastric lesions [27]. Pretreatment with 100 or 300 mg/kg IFE significantly attenuated the gastric lesions by 90.7% and 98.7%, respectively, compared with the HCl/ethanol-treated control group. The gastroprotective effects of 300 mg/kg IFE were similar to those of Stillen administered at the same dose. In histological observations of hematoxylin and eosin (H&E) staining, an improvement to the histopathological changes, such as disruption and exfoliation of the gastric mucosal epithelium, was observed by pretreatment with IFE, which supported the gastroprotective effects of IFE. These results suggest that IFE may have gastroprotective effects against acute gastric mucosal injury.

Gastric wall mucus is secreted by mucous neck cells and forms a thick layer covering the gastric mucosa [28]. The increased mucus acts as a barrier against hydrogen ion diffusion and enhances the buffering effects of gastric juices, thereby inhibiting gastric ulcer formation [29,30]. Ethanol-induced gastric mucus depletion is one of the pathological mechanisms involved in the development of gastric ulcers [31]. Pretreatment with 100 and 300 mg/kg IFE significantly inhibited the reduction in gastric wall mucus contents in the gastric mucosa by HCl/ethanol administration. These results suggest that IFE inhibits gastric mucosal damage by preventing the depletion of gastric wall mucus in gastric ulcers.

The gastric mucosa maintains its function and structure through a balance between aggressive and protective factors [32,33]. Increased levels of aggressive ulcerogens result in the overproduction of ROS, such as superoxide anions, hydroxyl radicals, and hydrogen peroxide, and the depletion of protective factors, such as antioxidants (SOD, CAT, GSH, etc.), causing gastric mucosal erosion and ulcers [34,35,36,37]. In rat gastric tissue, SOD and CAT activities and GSH levels decrease by one-third to one-half after HCl/ethanol administration, compared with normal rats, and inhibition of this decrease exerts gastroprotective effects [38,39]. SOD converts superoxide radicals into hydrogen peroxide and CAT converts hydrogen peroxide to water and oxygen [40]. GSH reacts non-enzymatically with superoxide, nitric oxide, hydroxyl radical, and peroxynitrite and functions as an ROS scavenger and a cofactor for GSH peroxidase in the neutralization of hydrogen peroxide [41]. MDA is produced by the peroxidation of cell membrane lipids and is widely utilized as an oxidative stress marker [42]. Herein, IFE significantly increased SOD and CAT activities and GSH levels and decreased MDA levels in ulcerated gastric tissues. In support of our results, *I. britannica* reportedly increases the levels antioxidant enzymes and exhibits free radical scavenging activity, and these effects are related to flavonoid components [14]. *I. britannica* extract and its flavonoid fractions enhance SOD and CAT activities and decrease MDA levels in the skin and liver tissues of rats [17,18,20]. In addition, several flavonoid compounds in *I. britannica* reportedly increase CAT activity and GSH levels in rat cortical cells [19]. Butanol fractions and flavonoid compounds from *I. britannica* show free-radical scavenging activity [43]. Along with these previous studies, our results indicate that IFE exerts gastroprotective effects by reducing oxidative stress.

IFE pretreatment significantly increased PGE_2_ levels in the gastric tissues of rats following the induction of gastric lesions by HCl/ethanol. Prostaglandins are expressed throughout the gastrointestinal tract and are involved in maintaining and protecting the gastric mucosa by modulating gastric blood flow, gastric acid and bicarbonate secretion, and gastric mucus production [44]. HCl/ethanol intake decreases PGE_2_ levels by one-fifth to one-third in rat gastric tissue [38,45], causing gastric ulcers and exacerbating existing gastric ulcers [46,47]. The administration of PGE_2_ or inhibition of PGE_2_ reduction inhibits gastric mucosal damage caused by ethanol [46]. Similar to previous results, these findings indicate that increased PGE_2_ levels might be involved in mediating the gastric mucus secretion of IFE.

This study had some limitations. First, it is difficult to draw a solid conclusion on whether the IFE re-established gastric mucosal homoeostasis, because a normal control group was not included in this study. Thus, it is necessary to confirm the gastroprotective effect of the IFE with a normal control group. Second, in this study, we used an HCl/ethanol-induced rat gastric ulcer model, which resembles gastric lesions caused by alcohol consumption. As gastric ulcers can also be induced by several factors, such as non-steroidal anti-inflammatory drugs, *H. pylori* infection, and ischemia [3,48], further studies are needed to investigate the effects of IFE in various ulceration models. Third, in this experiment, as the low dose of IFE, i.e., 100 mg/kg, had a high gastroprotection effect (90.7%); thus, further studies are needed to determine the lowest effective dose. Finally, we did not identify the active components of IFE for gastroprotection against HCl/ethanol-induced ulceration. However, it is known that sesquiterpene lactones and flavonoids, the main compounds of *I. Britannica*, are responsible for its various pharmacological effects [14]. In particular, 1-*O*-acetylbritannilactone, the major sesquiterpene lactone in *I. britannica*, exerts gastroprotective effects in caffeine-arsenic ulcers [49], and flavonoid compounds are responsible for the antioxidant effects of *I. britannica* [19]. Based on these previous findings, sesquiterpene lactones and flavonoids might be responsible for the gastroprotective effects of IFE, and further studies are needed to address this limitation.

## 4. Materials and Methods

### 4.1. Plant Material

Dried Inulae Flos was obtained from Young Chang Medicinal Herbs Co. (Seoul, Korea). The raw materials were authenticated by Professor Hocheol Kim of the College of Korean Medicine, Kyung Hee University. The voucher specimen (no. 191204004) was deposited in the Herbarium of NeuMed Inc. (Seoul, Korea).

### 4.2. Preparation of Sample Extracts

Dried flowers were ground and extracted, using a reflux apparatus, for 3 h, with 10 volumes of 95% ethanol, at 80 °C. The extract was filtered and concentrated under reduced pressure, using a rotary vacuum evaporator. The concentrated extract was then freeze-dried to obtain a powdered extract (extraction yield: 6.67%). The powdered extract was stored at 4 °C until use.

### 4.3. HPLC Analysis

The content of 1-O-acetylbritannilactone, the major active compound of Inulae Flos [14], was analyzed by using an Agilent 1220 Infinity HPLC system, including a G4281B binary pump, G4282B autosampler, and G4285B diode array detector (Agilent, Santa Clara, CA, USA). A Sunfire C_18_-column (250 × 4.6 mm id, 5 µm particle size; Waters, Milford, MA, USA) was used at room temperature. The separation was performed by reverse-phase gradient elution, using a mobile phase of 0.1% phosphoric acid (A) and acetonitrile (B) at a flow rate of 1.0 mL/min. The linear gradient was as follows: 0–15 min, 20–20% B; 15–40 min, 20–70% B; 40–45 min, 70–70% B; 45–50 min, 70–20% B; and 50–55 min, 20–20% B. The content of 1-O-acetylbritannilactone in the sample was quantified by measuring the peak areas at 210 nm and comparing them with a standard compound.

### 4.4. Animals

Six-week-old male Sprague-Dawley rats were purchased from Samtako Inc. (Gyeonggi-do, Korea) and acclimated for 1 week before use. Rats were housed under a controlled temperature (23 ± 1 °C), relative humidity (55% ± 5%), and light/dark cycle (12/12 h). Rats were allowed ad libitum access to food and water during the acclimatization period and fasted overnight the day before gastric lesion induction. All experimental procedures were approved by the Institutional Animal Care and Use Committee of the Korea Institute of Science and Technology for Eastern Medicine (approval no. KISTEM-IACUC-2018-001).

### 4.5. HCl/Ethanol-Induced Gastric Injury

Acute gastric ulceration was induced by HCl/ethanol administration according to Mizui and Doteuchi’s method [50]. Rats were randomly divided into four groups: control, Stillen 300 mg/kg (positive control), IFE 100 mg/kg, and IFE 300 mg/kg. Stillen (Dong-A ST Co., Ltd., Seoul, Korea), an ethanol extract of *Artemisia asiatica*, has been approved for gastritis treatment in Korea and has been shown to exert gastroprotective effects against various inducers [25]. Doses of IFE 100 and 300 mg/kg were selected based on the traditional dose range of Inulae Flos in Asian folk medicine. As the aim of this study was to provide a scientific basis for traditional use, we converted the daily human consumption to rats, based on body surface area [51]. The daily human dose of Inulae Flos ranges from 12 to 100 g/day [52,53]. Given the extraction yield and body surface area, the rat doses of 100 and 300 mg/kg/day correspond to human doses of 15 and 45 g/day. IFE or Stillen was orally administered at a volume of 10 mL/kg body weight after overnight fasting. The control rats were administered distilled water (vehicle), using the same regimen. One hour after treatment, 1.5 mL of 150 mM HCl/60% ethanol solution was orally administered, and, 1 h later, rats were sacrificed by cervical dislocation under isoflurane anesthesia. The stomach of each rat was removed quickly and incised along the greater curvature.

This experiment was conducted twice. First, 24 rats (six per group) were used to measure the gastric lesion index, histopathology, antioxidant enzyme levels, and PGE_2_ levels. Next, 32 rats (eight per group) were used to determine gastric wall mucus contents. In the first experiment, the stomach was washed with sterile saline and divided into right and left halves, which were stretched on a plate and photographed, to measure the lesion area. Then, the glandular region of the half was immersed in 4% paraformaldehyde solution, at 4 °C, for histopathological examination, and the glandular region of the remaining half was stored at −80 °C, for the measurement of antioxidant enzymes and PGE_2_.

### 4.6. Measurement of Gastric Lesion Index

The areas of the stomach and lesions were measured by using ImageJ (version 1.53d; National Institutes of Health, Bethesda, MD, USA). The gastric lesion index was calculated as follows:

$$\mathrm{Gastric}\;\mathrm{lesion}\;\mathrm{index}=\;\frac{Gastric\;lesion\;area\;\left(mm^{2}\right)}{Total\;gastric\;area\;\left(mm^{2}\right)}\times 100$$

### 4.7. Histopathological Examination of the Gastric Mucosa

Fixed gastric tissues were embedded in paraffin blocks and sectioned to a thickness of 4 µm. Tissue sections were stained with H&E, using a staining kit from ScyTek Laboratories Inc. (cat. no. HAE-1; West Logan, UT, USA) according to the manufacturer’s instructions, and observed by using a light microscope (Nikon Eclipse Ci-L; Nikon Corp., Tokyo, Japan).

### 4.8. Measurement of SOD and CAT Activities and GSH and MDA Levels

Frozen gastric tissues were ground and homogenized in 50 mM sodium phosphate buffer (pH 7.0) with 1 mM ethylenediaminetetraacetic acid (EDTA). Homogenates were centrifuged at 10,000× *g* for 15 min, at 4 °C, and the supernatants were collected for analyses. SOD and CAT activities and GSH levels were determined by using commercial kits (cat. nos. 706002, 707002, and 703002, respectively; Cayman Chemical Co., Ann Arbor, MI, USA) according to the manufacturer’s instructions. Enzyme activities and GSH levels were normalized to the wet weight of the stomach. MDA levels were measured by a lipid peroxidation (MDA) assay kit (cat. no. MAK085; Sigma-Aldrich, St. Louis, MO, USA), following the manufacturer’s instruction, and normalized to the wet weight of the stomach.

### 4.9. Quantification of PGE_2_ Concentrations

Frozen gastric tissues were ground and homogenized in 100 mM phosphate buffer (pH 7.4) with 1 mM EDTA and 10 μM indomethacin. Homogenates were centrifuged at 8000× *g* for 10 min, at 4 °C, and the supernatants were collected for analyses. The concentration of PGE_2_ was measured by using a commercial enzyme-linked immunosorbent assay kit (cat. no. 500141; Cayman Chemical Co., Ann Arbor, MI, USA) according to the manufacturer’s instructions. The concentrations were normalized to the wet weight of the stomach.

### 4.10. Determination of Gastric Wall Mucus Content

The gastric wall mucus content was measured by using the Alcian blue method. The glandular region of the stomach was rinsed with 0.25 M sucrose, immersed in 0.1% (*w*/*v*) Alcian blue solution for 2 h, and washed twice with 0.25 M sucrose for 15 min. The Alcian blue dye complexed with mucus was eluted by immersion in 15 mL of 0.5 M MgCl_2_ solution for 2 h, with horizontal shaking every 30 min. Then, the solution was shaken with an equal volume of diethyl ether and left to stand for 10 min. The lower aqueous phase was collected, and the absorbance was measured at 605 nm, using a spectrophotometer (Epoch 2; BioTek, Winooski, VT, USA).

### 4.11. Statistical Analysis

All values are expressed as means ± standard deviations. One-way analysis of variance, followed by the Dunnett’s test, was performed by using GraphPad Prism 8 (GraphPad Software Inc., La Jolla, San Diego, CA, USA). Results with *p*-values of less than 0.05 were considered statistically significant.

## 5. Conclusions

In conclusion, our findings suggest that Inulae Flos has strong gastroprotective effects, which are related to the enhancement of antioxidant activity and inhibition of gastric mucus depletion. Based on these results, Inulae Flos may be a promising agent for the prevention and treatment of gastritis and gastric ulcers.

## Figures and Tables

**Table 1:** Effects of IFE on gastric lesion area and index in HCl/ethanol-induced gastric ulcerated rats.

Groups	Gastric Lesion Area (mm^2^)	Gastric Lesion Index	Inhibition (%) ^1^
Control	64.22 ± 38.20 ^2^	7.90 ± 4.81	-
Stillen 300 mg/kg	1.09 ± 1.24 ***	0.13 ± 0.14 ***	98.35
IFE 100 mg/kg	5.97 ± 6.13 ***	0.43 ± 0.61 ***	94.56
IFE 300 mg/kg	0.86 ± 1.27 ***	0.10 ± 0.15 ***	98.73

^1^ Inhibition rate against the gastric lesion index. ^2^ Values are expressed as means ± standard deviations (*n* = 6/group). *** *p* < 0.001 versus the control group by one-way analysis of variance with post hoc Dunnett’s test. IFE, Inulae Flos extract.
